# A Study to Evaluate Mobile Phone Dependence Among Students of a Medical College and Associated Hospital of Central India

**DOI:** 10.4103/0970-0218.66878

**Published:** 2010-04

**Authors:** Sanjay Dixit, Harish Shukla, AK Bhagwat, Arpita Bindal, Abhilasha Goyal, Alia K Zaidi, Akansha Shrivastava

**Affiliations:** Department of Community Medicine, MGM Medical College, Indore - 452 001, Madhya Pradesh, India

## Introduction

Nomophobia(1) literally means no mobile phobia that is the fear of being out of mobile phone contact. If a person is in an area of no network, has run out of balance or even worse run out of battery, the persons gets anxious, which adversely affects the concentration level of the person. In recent times there seems to have been a transformation of the cell phone from a status symbol to a necessity because of the countless perks that a mobile phone provides like personal diary, email dispatcher, calculator, video game player, camera and music player.(2) Indian market has emerged as the second-largest market after China for mobile phone handsets. Our study was undertaken to find out the prevalence of nomophobia in the Indian scenario considering the tremendous increase in the number of mobile phone users in the past decade. We decided to conduct the study in our college since the younger generation is the latest consumer of the mobile phones, and the under 25 year age group in professional colleges like medical colleges use mobile phones quite frequently since most of them reside in hostels. Day scholar students too want to be in constant touch with their family members and friends since they are out of their homes for the whole day and at nights while studying in colleges and working in hospitals.

## Materials and Methods

The present study was a cross-sectional study conducted amongst 200 M.B.B.S. students from M.G.M. Medical College, Indore. Initially, students from different batches and those pursuing internship, both day scholars and hostlers, using mobile phone for more than one-year duration for at least 1-2 h per day were included for the study. Six hundred students of the college met the above inclusion criteria of which every third student was selected by systematic random sampling. A pre-designed and pre-tested questionnaire(3) designed on the lines of one developed by Dr. Marcus L. Raines(4) was used to study mobile phone dependence among the study subjects. The questionnaire was modified according to the local conditions. The mobile phone dependent students were then designated as nomophobes.

The data were collected to elicit information on demographic and psychographic aspects of the respondents. The demographic variables included age, gender, education and residence. The psychographic variables included attitude towards usage of cellular phones, mobile phones dependence and associated anxiety. The questionnaire focusing on nomophobia had eight components: duration of having mobile phone with self; anxiety and stress experienced because of faulty connections; loss of mobile and battery discharge; amount spent per month on recharge (options expressed in Rupees as compared to the original questionnaire that had options in Pounds); reaction shown to phone ringing at inappropriate times; frequency of change of phone/sim cards (the original questionnaire had an option focusing on termination of contract that was modified to change of phone every one to two years as India has an extensive network of prepaid subscribers who can change their phones and sim cards as and when required) and reactions because of inability of using the phone for a period of one week. Every question was compulsory and consisted of three options depicting maximum to minimum mobile phone association. The changes made according to local needs were pretested. The individual responses thus obtained were then compiled, processed and analysed to arrive at the results on various issues. The available online questionnaire analyses the scores of each respondent: it uses a cut-off limit of 24 for designating an individual as nomophobe; individuals with scores from 20 to 24 are considered at risk.(4) The data of the individual in the study were fed into the questionnaire available online(4) and scores were generated for each study subject. The nomophobes and at risk individuals were thus ascertained for individuals participating in the study. Involvement of students in sports and social activities was not included in the study.

## Results

The study population comprised 106 (53%) males and 94 (47%) females; of these 92(46%) were day scholars and 108 (54%) were residents of hostels. The majority of students were of the age group 17-28 year, of which 80 (40%) were of 20 year of age. Out of the entire study group, the students having nomophobia score 10-23 were maximum from I ^st^ professional (20.5%) and least from III ^rd^ professional -part - 2 (8%). The students having nomophobia score >24 were maximum from III ^rd^ professional part - 1 (7%) and minimum from internship (1%). Overall, 18.5% students were found to be nomophobes. In gender-based observation, 19% males and 18% females were found to be nomophobes. Twenty one out of 109 (19.26%) hostellers and 16 out of 91 (17.58%) day scholars were found to be nomophobic. No statistically significant association was observed in relation to gender, place of stay and academic sessions with nomophobia score. Approximately 73% students responded that they keep their mobile phones with them even when they go to sleep (for 24 h a day), 18.5% students used mobile phone during college hours and 8.5% students used it whenabsolutely necessary; 20% students responded that they lose their concentration and become stressed when they do not have their mobile around or their mobile has run out of battery; 44% students responded that they spend Rs. 250-500 per month for their mobile recharge; 25% students said that they upgrade their mobile software at least once a year; 83% students responded that mobile phone is a necessary tool to help them keep connected with their family members; 38.5% students responded they keep on checking their mobile phones for messages and calls; 31% students have at least one long duration call everyday for more than 30 min of which 39% comprised of females and 24% males. About 56% students kept their mobile phones either in the pocket of shirt or jeans close to their body so that they can have a feel of constant touch with their mobile phone. While attending classes or hospital duties, 23% 1 ^st^ Prof. students, 15% 2 ^nd^ Prof Jr. batch, 11% 2 ^nd^ Prof. Sr. batch, 25% 3 ^rd^ Prof Part-1, 10% 3 ^rd^ Prof Part-2 and 13% interns kept their mobile phones on silent mode or switched off while 1% students of 1 ^st^ Prof, 2 ^nd^ Prof Jr. and Sr. batch, 3 ^rd^ Prof Part-1 and Part-2 and 4% interns answered immediately even while attending classes or during duty hours [Tables 1 and 2].

**Table 1:** Nomophobia scores of the respondents based on the grade/year in which they study

Nomophobia Score	1^st^ Prof.	2^nd^ professional	2^nd^ professional	3^rd^ professional	3^RD^ professional	Internship	Grand Total
10–23	41 (20.5)	24 (12)	17 (8.5)	38 (19)	16 (8)	27 (13.5)	163 (81.5)
>24	05 (2.5)	07 (3.5)	04 (2)	14 (7)	05 (2.5)	02 (1)	37 (18.5)*
Grand Total	46 (23)	31 (15.5)	21 (10.5)	52 (26)	21 (10.5)	29 (14.5)	200 (100)

* It shows that around 19% students are nomophobes, Figures in parenthesis are percentages

**Table 2:** Nomophobia scores of the respondents based on gender

Nomophobia Score	Females	Males	Grand Total^*^
10–23	77 (81.9)	86 (81.13)	163 (81.5)
>24	17 (18.1)	20 (18.86)	37 (18.5)
Grand Total	94 (100)*	94 (100)*	200 (100)

* It shows that 19% males and 18% females suffer from nomophobia, Figures in parenthesis are percentages

## Discussion

At present there is not much information about the topic. According to the study the sample screened consisted of 53% males and 47% females of which 18.5% were found to be nomophobic. The result of the study shows that this disorder is equally prevalent among the study group irrespective of gender.

A study from United Kingdom on 2163 people revealed that 53% of the subjects tend to be anxious when they lose their mobile phone, run out of battery or credit or have no network coverage. The study found that about 58% of men and 48% of women suffer from the phobia, and an additional 9% feel stressed when their mobile phones are off. About 55% of those surveyed cited keeping in touch with friends or family as the main reason that they got anxious when they could not use their mobile phones.(1) A study conducted by Market Analysis and Consumer Research Organization (MACRO) in Mumbai to study the various patterns and association of mobile phone usage reported that 58% of the respondents could not manage without a mobile phone even for a day.(2)

The present observations in this study are from a small group of students only, which may not reflect the scenario worldwide since millions of cellular mobile subscribers are added every month indicating that full blown nomophobia has all the possibilities to reach to the epidemic scale. In reality these results give an alarming indication that as days goes by the youth is getting more and more dependent on mobile phones, which may lead to serious psychiatric(5) and psychological problems among the users. To avoid the stress induced because of malfunctioning of mobile phones, people using it should carry a charger all the time, prepaid phone card to make emergency call in case their mobile is not functioning, credit balance in their mobile, should supply family members and friends alternate contact number and store important phone numbers somewhere else as backup in the case they lose their mobile phone.(6) People should assess their addiction with mobile phone (nomophobia status) with the help of online teaching sites, which can help reduce the anxiety levels(7) because of mobile phone overuse.

## Conclusion

The results of the study are suggestive of mobile phone dependence among students of M.G.M. Medical College, Indore. The data is indicative of nomophobia to be an emerging problem of the modern era. Multicentric studies are required to assess the real problem and thereby take appropriate steps to tackle the growing problem.
